# Effectiveness of personalized smoking cessation intervention based on ecological momentary assessment for smokers who prefer unaided quitting: protocol for a randomized controlled trial

**DOI:** 10.3389/fpubh.2023.1147096

**Published:** 2023-07-31

**Authors:** Min Jin Zhang, Wan Jia Aaron He, Tzu Tsun Luk, Man Ping Wang, Sophia Siu Chee Chan, Yee Tak Derek Cheung

**Affiliations:** School of Nursing, LKS Faculty of Medicine, The University of Hong Kong, Hong Kong, Hong Kong SAR, China

**Keywords:** ecological momentary assessment (EMA), instant messages, mobile health (mHealth), smoking cessation, telephone counseling, unaided quitting

## Abstract

**Introduction:**

Ecological momentary assessment (EMA)-based smoking cessation intervention may help personalize intervention for smokers who prefer to quit smoking unaided. This study aims to evaluate the effectiveness of EMA-based phone counseling and instant messaging for smoking cessation.

**Methods/design:**

This is a two-arm, accessor-blinded, simple individual randomized controlled trial (allocation ratio 1:1). Participants will be recruited from community sites and online platforms in Hong Kong. Interventions will be delivered via a phone call and instant messaging. Current adult smokers who (1) self-report no intention to use smoking cessation services and medication in the coming month and (2) have not used smoking cessation services or nicotine replacement therapy in the past 7 days will be recruited. Recruited participants will be randomized to intervention or control groups via an online randomizer. All participants will be required to complete EMAs (five times per day for 7 consecutive days). The intervention group (*n* = 220) will receive a nurse-led brief phone counseling immediately after the 1-week EMAs and 10-week EMA-based advice via instant messaging applications (e.g., WhatsApp, WeChat). The 10-week EMA-based advice covers a summary of the 1-week EMAs, and tailored cessation support focused on personalized smoking triggers. The control group (*n* = 220) will not receive any intervention during the same period. The primary outcomes are participants' progression toward smoking cessation assessed by the Incremental Behavior Change toward Smoking Cessation (IBC-S) and biochemically validated abstinence at the 3-month follow-up. Secondary outcomes include self-reported and biochemically validated tobacco abstinence at the 6-month follow-up.

**Discussion:**

The findings will provide evidence that the EMA-based tailored smoking cessation intervention can be adapted as a new health promotion strategy for current smokers who are unwilling to use smoking cessation aids.

**Clinical trial registration:**

https://classic.clinicaltrials.gov/ct2/show/NCT05212220, identifier: NCT05212220.

## Introduction

Smoking is the leading modifiable risk factor for premature death (1). Health risks associated with smoking, such as heart disease, stroke, and lung cancer, decrease with increasing years of abstinence (2). Proven behavioral and pharmacological smoking cessation interventions can double the chance of successful quitting (3–5). However, globally, unassisted quitting is still the major method for quitting. For example, a systematic review conducted in Australia showed that 54%−78% of ex-smokers quit unassisted, and 41%−82% of current smokers had attempted to quit unassisted (6). Unassisted quitting is also prevalent in Asian countries. A population-based survey of male smokers in China showed that 87.6% of those who made a quit attempt did so unaided, while 97% of ex-smokers successfully quit without any assistance (7). Another randomized controlled trial (RCT) conducted in Hong Kong found that only 3.4% of community daily smokers utilized smoking cessation services 6 months after receiving brief cessation advice and a self-help booklet (8). Barriers for current smokers seeking cessation services include a lack of interest in quitting, low accessibility (e.g., work and time constraints, a lack of information, and perceived availability), a reluctance to disclose personal information to the service providers, and a lack of social support (9, 10). In addition, the culture of self-reliance in solving problems makes Chinese smokers less likely than Western smokers to seek cessation services (11). Therefore, there is a need for new modes of delivering smoking cessation to overcome these barriers.

Ecological momentary assessment (EMA) is the self-administered documentation of real-time data on real-world behaviors, cognitions, or events (12). EMA aims to study the behavioral processes close to the moment of assessment or over the very recent times, which can reduce recall bias and social desirability bias in reporting pervasive and negative behaviors (12, 13). EMA can collect data at multiple time points in the natural environment, which is particularly useful for measuring addictive behaviors such as smoking (12). Smoking is a discrete and repeatable event driven by emotional triggers (e.g., stress, anxiety, boredom) (14), habitual triggers (e.g., drinking alcohol, finishing a meal) (15), social triggers (e.g., attending a bar, being with friends/others who are smoking) (16, 17), and withdrawal symptoms (e.g., irritability and trouble sleeping) (18). Previous EMA studies have identified exposures to prior smoking triggers, including exposure to secondhand smoking (17), proximity to tobacco retail outlets (17), and negative affect (19). Thus, EMA data can facilitate a better understanding of how smoking triggers influence tobacco consumption in smokers.

EMA-based smoking cessation intervention has been defined as tailored treatments provided to smokers based on individual smoking behaviors and smoking triggers obtained from EMAs over a certain period in the real-world environment. The interventions are delivered after the completion of all EMAs. Ecological momentary intervention (EMI), which is an intervention delivered immediately after each episode of EMA, has been used in smoking cessation in previous studies (20, 21). The first EMI trial in the United States found that delivery of tailored messages immediately after each EMA episode could significantly reduce the smoking urge in current smokers who are willing to quit within 7 days (20). However, EMI has some limitations. First, EMI normally has an algorithm to automatically and immediately send appropriate messages in response to the users' input. Automatically sending personalized messages can save manpower from processing, but interaction with real people can be crucial for psychosocial support (22). Second, EMI pushes instant messages continuously after users complete the EMA. However, such high-intensity intervention may not be suitable for smokers who have no intention to quit, as they are less likely to accept these immediate responses (23). While smokers who are not ready yet to quit might not proactively seek smoking cessation aids, they may be more interested in using mHealth to monitor smoking behaviors and receive support when required (23). Hence, developing an EMA-based personalized quit plan, which provides personalized cessation intervention after all EMAs, is more appropriate for smokers who are not ready to receive smoking cessation services.

In Hong Kong, where the smoking prevalence was low (10.3% in 2021), only 3.8% of current smokers have ever used a smoking cessation service (24). Thus, encouraging smokers to use current smoking cessation services remains a challenge. Therefore, new and effective strategies are needed to motivate smoking cessation and the greater use of smoking cessation services. This RCT aims to evaluate the effectiveness of EMA-based smoking cessation interventions for quitting preparation and tobacco abstinence in the past 7 days at the 3-month follow-up. We will target smokers who do not intend to use smoking cessation services; therefore, in addition to tobacco abstinence, our intervention has another goal, i.e., to motivate them to prepare for quitting.

## Methods

### Study design

**Figure 1:**
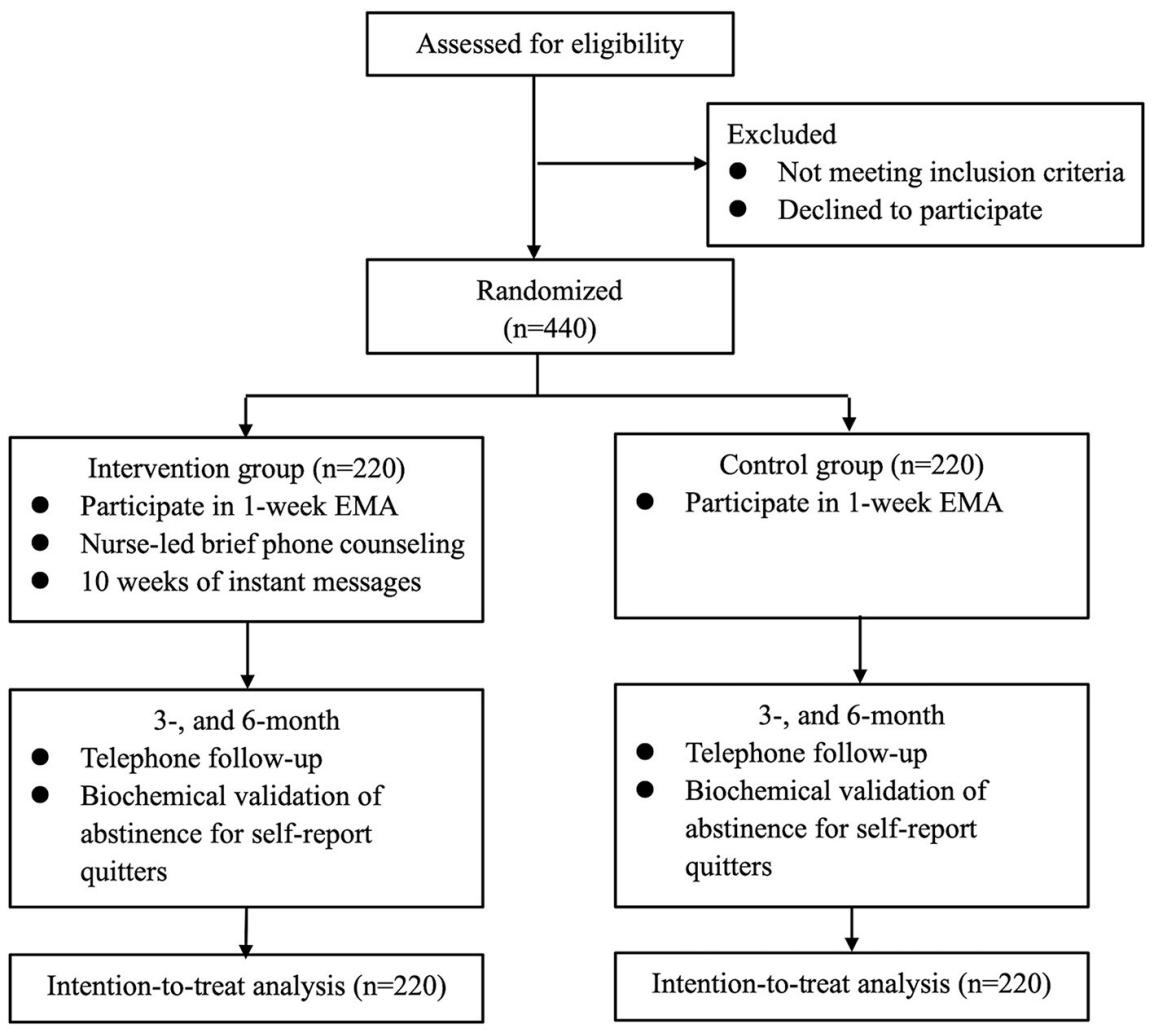
Study flowchart.

This is a two-arm RCT (allocation ratio 1:1) nested within an EMA-based observational study. During the 1-week EMA, all participants will be prompted to complete EMAs five times per day for 7 consecutive days to document their smoking triggers, smoking behaviors, and daily cigarette consumption. Subsequently, the intervention group will receive an EMA-based intervention, including 15-min nurse-led brief phone counseling and 10-week tailored messages via instant messaging applications. The control group will not receive any intervention after the 1-week EMA. Both groups will be followed up at 3 and 6 months (see Figure 1). The Consolidated Standards for Reporting of Trials (CONSORT) criteria will be followed. The research protocol has been registered with ClinicalTrials.gov (NCT05212220).

### Ethic

This study has obtained ethical approval from the Institutional Review Board of the University of Hong Kong, Hospital Authority Hong Kong West Cluster (UW 20-524). Online consent to participate in this study will be obtained from study participants.

### Participants

Smokers with the following inclusion criteria will be invited to participate in the trial: (1) daily smokers using traditional cigarettes, electronic cigarettes, or heated tobacco products in the past week; (2) age ≥18 years; (3) owning a smartphone with internet access; (4) those who will be staying in Hong Kong during the 1-week EMA study because the EMA application's prompting capability can only be used in Hong Kong due to technical restrictions; (5) those who are able to read and write Chinese; (6) those who have no plan to use smoking cessation services or medications in the coming month; and (7) those who have not used smoking cessation services or nicotine replacement therapy (NRT) in the past 7 days. Participants' smoking status will be confirmed by measuring exhaled carbon monoxide (≥4 ppm) using a Smokerlyzer or salivary cotinine (≥30 ng/ml) using an iScreen OFD Cotinine Saliva Test Device (25, 26). Smokers who (1) are pregnant or (2) are diagnosed with mental illness by psychiatrists will be excluded from this study.

### Recruitment procedures

The recruitment process will be conducted both offline and online. We will use a “foot-in-the-door” approach for offline recruitment, which has been shown to be a feasible and efficacious way to reach smokers at outdoor smoking hotspots (public outdoor areas where smokers gather to smoke) in the community in our previous study (27). Our recruitment staff will first approach hotspots and distribute free items (e.g., a tissue pack) to smokers. If the smoker is willing to accept free items and talk to the recruitment staff, the staff will ask further questions related to eligibility (28). Eligible participants will be given more details about this project and invited to provide online consent, after which they will receive assistance in installing the EMA application on their smartphone and completing the baseline questionnaire [either on a paper form or online form (Qualtrics, Provo, UT)]. Due to the time limit for outdoor recruitment, participants can complete the baseline questionnaire after the recruitment session.

We will also send mass emails or post advertisements online (e.g., Facebook, Instagram, or Yahoo!). Potential participants interested in this project can complete the online application form (via Qualtrics) by scanning the Quick Response code or clicking the URL on the advertisement. Our recruitment staff will then make appointments with the potential participants to complete recruitment procedures via face-to-face or virtual meetings on Zoom. During the face-to-face recruitment meeting, our recruitment staff will instruct them to do the saliva test to confirm their smoking status, complete the online consent form and the baseline questionnaire, and install and set up the EMA application. If participants prefer a virtual meeting, we will first mail them the saliva test kit. They will probably receive the kit in 2–3 days. During the virtual recruitment meeting, the recruitment staff will instruct the potential participant to verify their smoking status using the saliva test kit. Only those who pass the saliva test will be invited for the subsequent recruitment procedure.

### EMA application

An EMA application has been developed for smokers to document all smoking triggers and smoking-related behaviors. After installing the EMA application on participants' smartphones, participants need to input the last five digits of their telephone number as an identifier and select the study start date (participants can start the next day at the earliest). Participants need to complete five signal-contingent EMAs per day for 7 consecutive days during their awake time, with a 3-h interval between two prompts. For example, if a participant sets the first prompt time at 9:00 a.m., he/she will be asked to complete an EMA at 9:00 a.m., 12:00 p.m., 3:00 p.m., 6:00 p.m., and 9:00 p.m., respectively.

In each signal-contingent EMA time window, the application will first prompt the user to answer questions on smoking triggers and smoking-related behaviors, which takes ~1 min. If the user does not respond to the EMA prompt within 5 min, two subsequent prompts will be generated within 10 min. The corresponding EMA will be regarded as non-response if these additional prompts are not answered. All data will be uploaded to the server located at The University of Hong Kong immediately after each EMA completion.

The EMA questions on smoking triggers cover four domains: emotional, social, habitual, and withdrawal triggers. Emotional triggers include three positive affects (PA; relaxation, happiness, and other PA) and six negative affects (NA; interpersonal conflict, loneliness, helplessness, boredom, worry, and other NA). Social triggers include social gatherings, going to entertainment venues, seeing others smoking, and being recommended tobacco products by others. Habitual triggers include during breaks, upon waking, after meals, and at work. Withdrawal triggers include exposure to secondhand smoke, depression, irritability, tension, anxiety, restlessness, fatigue, and poor concentration (29). Smoking-related behaviors include (1) whether you have consumed tobacco (traditional cigarettes, heated tobacco products, or electronic cigarettes) in the past 3 h and the number of cigarettes consumed; (2) whether you have had tobacco urges in the past 3 h and types of craving (physical, psychological, or social craving); and (3) whether you have purchased tobacco products in the past 3 h. If smoking-related behaviors and smoking triggers are reported in the same EMA survey, the application will prompt users to state if they are associated. In the first EMA of each day, sleep quality will be assessed by the following questions: (1) Did you experience insomnia problems last night (difficulty falling asleep, difficulty staying asleep, problems waking up too early) (score ranges from 0 (*none*) to 4 (*very severe*))? (2) How satisfied were you with your last night sleep pattern [score ranges from 0 (*very satisfied*) to 4 (*very dissatisfied*)]? The time for the first cigarette after waking up will be asked at the second EMA daily. Exposure to tobacco control policy, including health warnings on their tobacco pack, point-of-sale tobacco displays, smoking hotspots, and daily tobacco consumption, will be asked in the fifth EMA daily (Table 1). A reminder will be delivered to participants whose compliance rate drops to 50% or below in the first 3 study days.

**Table 1:** Questions in the five EMAs of a day.

	**First EMA**	**Second EMA**	**Third EMA**	**Fourth EMA**	**Fifth EMA**
**Smoking triggers**					
**Emotional triggers**					
Positive affect (PA): relaxation, happiness, and other PA	✓	✓	✓	✓	✓
Negative Affect (NA): interpersonal conflict, loneliness, helplessness, boredom, worry, and other NA	✓	✓	✓	✓	✓
**Social triggers**					
Social gatherings, going to entertainment venues, seeing others smoking, and being recommended tobacco products by others.	✓	✓	✓	✓	✓
**Habitual triggers**					
During breaks, upon waking, after meals, and at work	✓	✓	✓	✓	✓
**Withdrawal triggers**					
Exposure to secondhand smoke, depression, irritability, tension, anxiety, restlessness, fatigue, and poor concentration	✓	✓	✓	✓	✓
**Smoking-related behaviors**					
Consumption of tobacco products (conventional cigarettes, electronic cigarettes, or heated tobacco products)	✓	✓	✓	✓	✓
Tobacco urge, types of craving (physical, psychological, or social craving)	✓	✓	✓	✓	✓
Purchasing tobacco products	✓	✓	✓	✓	✓
**Sleep quality**					
Sleep problems (difficulty falling asleep, difficulty staying asleep, and problems waking up too early)	✓				
Satisfaction toward last night sleep pattern	✓				
**Time to consume the first cigarette after waking**		✓			
**Tobacco control policy**					
Exposure to health warnings on their tobacco pack					✓
Exposure to point-of-sale tobacco displays					✓
Exposure to scenes of others' smoking					✓
**Daily tobacco consumption**					✓

Overly frequent EMAs may increase participant burden and decrease the completion rate, but too few EMAs and longer measurement intervals may disengage the participant from EMA and make them forget to follow the EMA protocol (30). A meta-analysis showed that, among substance users, a higher average compliance rate was observed for 4–5 EMAs per day (76.4%) compared to fewer 2–3 EMAs (69.8%) or more than 6+ EMAs (76.2%) (31). Furthermore, participants tend to become fatigued and lose motivation to complete EMA as the time of the EMA period increases, and the compliance rate declines as the EMA period increases beyond 1 week (32). Hence, five EMAs a day for 7 consecutive days applied in the present study are appropriate. Furthermore, establishing a strong sense of collaboration between research staff and participants is essential for a high response rate (33). From EMA records, smokers can learn more about their smoking behavior and triggers (e.g., nicotine withdrawal, stress, depression) (23). To help smokers learn from their EMAs, a brief phone counseling session can summarize smoking behaviors from 1-week EMAs, provide tips on how to cope with individualized smoking triggers, and provide a personal quit plan. The updated Cochrane systematic reviews found that adding text messaging interventions to other smoking cessation interventions (e.g., counseling) was more effective than those interventions alone (4, 34). Therefore, instant messaging and a nurse-led brief phone counseling will be used as interventions.

An incremental incentive strategy will be used. All participants will be given a HK$50 (US$6.37) shopping voucher for the completion of the baseline survey and a HK$15 (US$1.91) shopping voucher for completing three time-based EMA surveys within each day. They will be further rewarded with a HK$10 (US$1.27) shopping voucher for completing five surveys each day. In addition, participants will be further rewarded with HK$25 (US$3.19) shopping vouchers for completing more than 80% of the 1-week EMA surveys. Self-reported quitters who complete biochemical validation at 3- or 6-month follow-up will obtain an additional HK$50 (US$6.37) shopping voucher. Therefore, each participant can get up to HK$300 (US$38.22) for completing all requirements.

### Intervention

#### EMA-based intervention

After the 1-week EMA, participants in the intervention group will receive an EMA-based intervention package, which includes a 15-min nurse-led brief phone counseling and 10-week tailored messages via instant messaging applications. The intervention content is based on the US clinical practice guidelines for smoking cessation (35) and the Smoking Cessation Information Kit (published by the Department of Health) (36). According to the theory of the Health Action Process Approach (37), our 10-week tailored messages focused on two stages: (1) preintentional motivation processes (increase risk perception of smoking, positive expectancies of quitting, and perceived self-efficacy of quitting) and (2) postintentional volition processes (maintain self-efficacy, make a quit plan, and provide resources to overcome coping barriers) (38). Meanwhile, we followed the self-determination theory (39) that participants can autonomously choose the treatments they want rather than being required by our intervention providers (40).

#### Brief phone counseling

Brief phone counseling will be provided by trained research nurses with 2 years of experience in smoking cessation counseling. These nurses have received a training session on an overview of the research project and the nurse report template. The research assistant will first summarize the 1-week EMA record in the nurse report for each participant and then send participants' nurse reports to the nurse. Afterward, the nurse will provide a brief phone counseling session that includes: (1) a summary of the 1-week EMA observation, including type and frequency of craving, nicotine dependence level, and pro-smoking triggers; (2) self-help techniques to manage craving and pro-smoking triggers; (3) increasing motivation to quit by raising awareness of the harms of smoking, providing tips for coping with triggers, and improving outcome expectancies. If the participants are interested in quitting, the nurse can refer them to current smoking cessation services (optional).

#### 10-week instant messaging

After the counseling, participants in the intervention group will receive personalized cessation messages through instant messaging applications (e.g., WhatsApp and WeChat) for 10 weeks. Participants can choose to respond or not to our messages (Appendix 1). The 10-week instant messages can be classified as core messages and additional messages. Core messages, including the summary of the 1-week EMA, benefits of quitting, tips for quitting, methods to cope with nicotine withdrawal symptoms/encouragement to quit, and use of smoking cessation services (23) will be sent to all participants in the intervention group. Based on EMA records, we will identify participants' three most common triggers of smoking/urging/purchasing tobacco and send additional messages addressing these triggers. We will also send one additional message to address the participant's craving type and two messages on improving sleep quality if participants are not satisfied with his/her sleep quality (score ≥2 on a scale of 0–4 for the question of “how satisfied are you with your last night sleep pattern?”). The control group will not receive any intervention message.

### Randomization, blinding, and allocation concealment

Simple individual randomization (1:1 allocation ratio) will be applied. Qualtrics' randomizer will be used for random allocation. This will be implemented by research staff not involved in the recruitment to achieve allocation concealment. After a participant consent to the trial, the non-recruiting research staff will access the randomizer and enter the individual participant's 5-digit identifier. With the randomizer feature in place, Qualtrics will display a page indicating to which group the participant was allocated.

Participants and the staff of intervention delivery cannot be blinded because of the behavioral nature of the intervention. However, outcome assessors and data analysts will not be involved in the recruitment and intervention delivery and are thus blinded to the group allocation (e.g., single-blinded design).

### Baseline assessment

Baseline assessment includes sociodemographic characteristics, smoking characteristics, intention to quit, history of quit attempts, exposure to health warnings on their tobacco pack/tobacco point-of-sale display/scenes of others' smoking, anxiety, depression (41), sleep quality (42) and Incremental Behavior Change toward Smoking Cessation (IBC-S) scale (43). Smoking characteristics include nicotine dependence level, which is measured by the Heaviness of Smoking Index (44), current use of tobacco products, the four domains of smoking triggers, types of craving (physical, psychological, or social craving), frequency of urge during the past 7 days [score range from 0 (*never*) to 5 (*always*), and level of urge during the past 7 days [score range from 0 (*not at all*) to 5 (*extremely*). IBC-S is a scale that can detect the effect of interventions on moving participants toward quitting attempts and preparation (43).

### Outcomes

All participants will be contacted by blinded outcome assessors via telephone follow-up after 3 and 6 months. Table 2 shows the schedule of data collection.

**Table 2:** Schedule of enrollment, intervention delivery, and data collection.

	**Study period**														

	**Enrollment**	**Allocation**	**Post-allocation**												
**Time point**	**–** *t*_1_	**0**	**W1**	**W2**	**W3**	**W4**	**W5**	**W6**	**W7**	**W8**	**W9**	**W10**	**W11**	**W12**	**W24**
**Enrolment**															
Eligibility screen	✓														
Informed consent	✓														
Allocation		✓													
Baseline assessment		✓													
**Interventions**															
**Intervention group**															
EMA			✓												
Brief phone counseling				✓											
**Instant messaging**					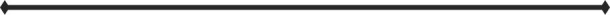										
**Control group**															
EMA			✓												
**Assessments**															
Sociodemographic characteristics		✓													
Smoking characteristics		✓													
Intention to quit		✓													
History of quit attempts		✓													
Exposure to health warnings on their tobacco pack/tobacco point-of-sale display/scenes of others' smoking		✓													
Sleep quality		✓												✓	✓
Anxiety/depression		✓												✓	✓
IBC-S		✓												✓	✓
Biochemically validated abstinence														✓	✓
Self-reported 7-day point-prevalence abstinence														✓	✓
Self-reported use of smoking cessation service or medication														✓	✓
Quit attempts														✓	✓
Self-efficacy on quitting		✓												✓	✓

#### Primary outcomes

The primary outcomes are IBC-S (43) and biochemically validated abstinence in the past 7 days at the 3-month follow-up. Biochemical validation will be performed using exhaled carbon monoxide (Smokerlyzer, <4 ppm) and salivary cotinine (iScreen OFD Cotinine Saliva Test Device, <30 ng/ml) (25, 26, 45). Self-reported quitters using NRT at the 3-month follow-up will be validated using exhaled carbon monoxide only (46). Self-reported quitters who refuse to have an exhaled carbon monoxide test due to the COVID-19 pandemic will be validated using a saliva cotinine test kit only. At the 3-month follow-up, only those who self-report abstinence in the past 7 days will be invited for the biochemical validation.

#### Secondary outcomes

Secondary outcomes include (1) self-reported 7-day point-prevalence abstinence at 3- and 6-month follow-ups; (2) biochemically validated abstinence at 6-month follow-up; and (3) self-reported use of smoking cessation services or medication from baseline at 3- and 6-month follow-ups. Ancillary outcomes include quit attempts at 3 and 6 months, satisfaction with the EMA and the study procedures, perceived importance (47), confidence (47), and difficulty (47) of quitting at 3 and 6 months. The nurse will document the content of the brief phone counseling in a report.

#### Process evaluation

For the process evaluation of the intervention, information on the type of intervention received, perceived usefulness of the intervention, and satisfaction toward the intervention will be measured at the 3-month follow-up. Brief phone counseling time will be collected from nurses. The EMA compliance rate and percentage of participants receiving both brief phone counseling and instant messages will also be calculated.

### Sample size

To date, there has been no similar RCT to provide an estimate of the effect size of the EMA-based intervention. An updated Cochrane network meta-analysis indicated that the odds ratio of biochemical validation abstinence between tailored short messaging service (SMS) focused on how to quit and minimal smoking cessation intervention was 1.92 (4). One of our previous pragmatic RCTs showed that the intention-to-treat (ITT) 6-month self-reported quit rate among Hong Kong current smokers without any intervention was 11% (48). To detect a significant difference in quit rate by using Fisher's exact test between two groups with a power of 80% (to reduce type II error) at a 5% significant level (type I error) calculated by G^*^Power software (version 3), a total of 452 participants (allocation ratio 1:1; 226 vs. 226) is needed. However, due to the limited budget for this project, we plan to recruit 440 participants in total.

### Statistical analysis

The primary analysis will examine the effect of the EMA-based intervention on biochemically validated abstinence at 3 months and the difference in the IBC-S score between the two groups. The ITT approach will be used to include all randomized participants in the denominators and assume non-respondents at the 3-month follow-up as smokers with no change in the IBC-S score. We will conduct logistic regression models to compare abstinence between the intervention and control groups. We will use multivariable linear and logistic regression models adjusted for imbalanced sociodemographics assessed at baseline to summarize the intervention effect on IBC-S and biochemically validated abstinence at 3-month follow-up. Multiple imputations will be used to impute missing data for the observed data as a sensitivity analysis. Another sensitivity analysis for the primary outcomes will be performed through a complete case analysis. Subgroup analysis of comparing the primary outcomes between intervention group participants who receive both nurse-led counseling plus instant messaging and instant messaging only will be conducted to assess the utility of the counseling. All statistical analyses will be conducted using Stata/SE (version 16) or R (version 1.3.1073).

### Current status

In total, 459 participants were recruited from 21 March 2022 to 3 January 2023. All follow-up and data collection are expected to be completed in July 2023.

## Discussion

It is the first RCT to examine the effectiveness of an EMA-based smoking cessation intervention on smoking cessation for current smokers. If the effectiveness of an EMA-based smoking cessation intervention is found, the intervention model can be adopted as a new health promotion strategy for engaging current smokers who do not want to use smoking cessation aids in quitting. Unlike most conventional smoking cessation studies, in which the target smokers are those who are motivated to quit with smoking cessation aids, our target smokers include those who have no intention to use smoking cessation aids within the following month and have not used smoking cessation aids in the past 7 days (24). Therefore, it is of great importance to develop a new mode of smoking cessation intervention for the majority of smokers in the community. Throughout the study, participants are not required to enroll or receive multiple types of counseling. They can still determine their quitting methods based on EMA-based self-monitoring and personalized advice.

## Ethics statement

This study has obtained ethical approval from the Institutional Review Board of the University of Hong Kong/Hospital Authority Hong Kong West Cluster (UW 20-524). Online written consent to participate in this study will be obtained from study participants.

## Author contributions

YTDC contributed to the conceptualization of the project. YTDC and MJZ contributed to the methods and investigation and wrote the original draft of the manuscript. MJZ served as the project administrator. All authors provided critical feedback and reviewed, edited, and approved the final manuscript.
