# Supplementary material for: Effectiveness of personalized smoking cessation intervention based on ecological momentary assessment for smokers who prefer unaided quitting: protocol for a randomized controlled trial
Source: Front Public Health. 2023 Jul 31;11:1147096. doi: 10.3389/fpubh.2023.1147096 (PMC10425238; doi:10.3389/fpubh.2023.1147096)
Supplement: Supplementary file 1 [file Table_1.DOCX]

Appendix 1. Example of instant messages

| 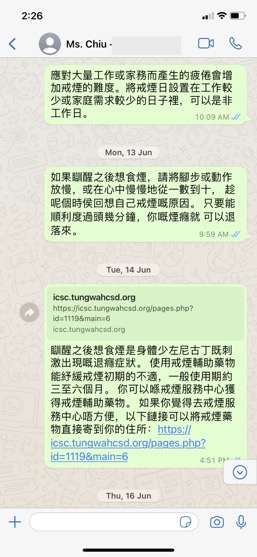 | | Tailored message  Fatigue from working or doing chores makes quitting smoking more difficult. When picking a date to quit, choose a date when you will have less work or family obligations, like a day off.  If you experience cravings in the morning, slow down your movements, count from 1 to 10 in your head, and think about your reasons for wanting to quit smoking. Your cravings will pass after the first few minutes.  Cravings in the morning when you wake up are a symptom of nicotine withdrawal. You can try nicotine replacement therapy (NRT) to reduce these symptoms that may occur the first few weeks after you quit. In general, you are recommended to use NRT for 3 to 6 months. You can obtain NRT from a smoking cessation clinic. If you are unable to visit a smoking cessation clinic, you can order NRT through the following link: <https://icsc.tungwahcsd.org/pages.php?id=1119&main=6> |
| --- | --- | --- |
| 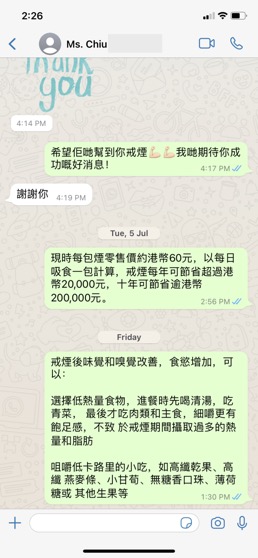 | | Regular message  I hope they will be helpful in your journey to quit smoking. We look forward to hearing about your success!  Thank you  A pack of cigarettes cost HKD$60. If you are smoking a pack a day, you can save more than HKD$20,000 a year by quitting smoking. In 10 years, you can save more than HKD$200,000.  When you quit smoking, you will notice an improvement in your sense of taste and smell, which may cause an increase in appetite. To avoid weight gain, you can:  Choose low-calorie foods, drink clear broths before your meal., eat vegetables before meat and carbs, and chew slowly for more satiety.  Chew on low-calorie snacks, such as high-fiber dried fruits, high-fiber granola bars, baby carrots, sugar-free gum, mint candy, and other fruits. |
| 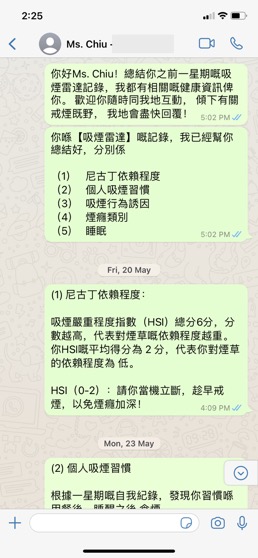 | Week 1 summary  Hello Ms. Chiu! Based on a summary of your responses in our app in the past week, we have prepared some health tips for you. You are welcome to interact and chat with us about anything quitting-related. We will try to get back to you as soon as possible!  We have acquired the following information based on your responses in our app.   1. Nicotine dependence level 2. Personal smoking habits 3. Personal smoking cues 4. Smoking craving type 5. Sleep quality   (1) Nicotine dependence level  The highest possible score on the Heaviness of Smoking Index (HSI) is 6. The higher your score, the more severe your dependence on nicotine. Your average HSI score was 2. This means you have a low dependence on nicotine.  HSI (0-2) : Make the decision to quit today to avoid being more dependent on smoking!  (2) Personal smoking habits  According to your week-long self-report records, you have a habit of smoking after eating and waking up in the morning. | |
| 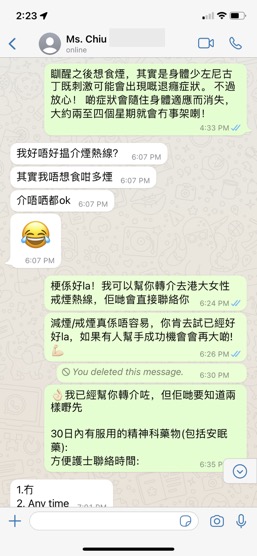 | | Interaction with P  Cravings in the morning when you wake up are a symptom of nicotine withdrawal, but don’t worry! These symptoms will subside as your body adjusts, and should be completely gone in 2 to 4 weeks.  Should I seek help from a smoking cessation hotline?  I don’t want to smoke so much  It’s okay even if I can’t quit completely  Of course! I can refer you to our school’s Women Quitline. They will contact you directly.  Quitting smoking is hard. The fact that you are willing to try is commendable. With professional help, your chances of quitting should also be higher!  I’ve already made the referral for you, but they need to know two things from you:  Have you taken any psychiatric medication (including Ambiens) in the past 30 days?  When would be most convenient for our nurses to contact you?  1. No  2. Any time |
